# A genome-wide scan for pleiotropy between bone mineral density and nonbone phenotypes

**DOI:** 10.1038/s41413-020-0101-8

**Published:** 2020-07-01

**Authors:** Maria A. Christou, Georgios Ntritsos, Georgios Markozannes, Fotis Koskeridis, Spyros N. Nikas, David Karasik, Douglas P. Kiel, Evangelos Evangelou, Evangelia E. Ntzani

**Affiliations:** 1 Department of Hygiene and Epidemiology, Clinical and Molecular Epidemiology Unit, School of Medicine, University of Ioannina, Ioannina, Greece; 2 Department of Medicine, Beth Israel Deaconess Medical Center, Harvard Medical School, Hinda and Arthur Marcus Institute for Aging Research, Hebrew SeniorLife, and the Broad Institute of MIT & Harvard, Cambridge, MA USA; 3 Azrieli Faculty of Medicine, Bar Ilan University, Safed, Israel; 4 Department of Epidemiology and Biostatistics, Imperial College London, London, UK; 5 Department of Health Services, Policy and Practice, Center for Research Synthesis in Health, School of Public Health, Brown University, Providence, RI USA

**Keywords:** Osteoporosis, Osteoporosis

## Abstract

Osteoporosis is the most common metabolic bone disorder globally and is characterized by skeletal fragility and microarchitectural deterioration. Genetic pleiotropy occurs when a single genetic element is associated with more than one phenotype. We aimed to identify pleiotropic loci associated with bone mineral density (BMD) and nonbone phenotypes in genome-wide association studies. In the discovery stage, the NHGRI-EBI Catalog was searched for genome-wide significant associations (*P* value < 5 × 10^−8^), excluding bone-related phenotypes. SNiPA was used to identify proxies of the significantly associated single nucleotide polymorphisms (SNPs) (*r*^2^ = 1). We then assessed putative genetic associations of this set of SNPs with femoral neck (FN) and lumbar spine (LS) BMD data from the GEFOS Consortium. Pleiotropic variants were claimed at a false discovery rate < 1.4 × 10^−3^ for FN-BMD and < 1.5 × 10^−3^ for LS-BMD. Replication of these genetic markers was performed among more than 400 000 UK Biobank participants of European ancestry with available genetic and heel bone ultrasound data. In the discovery stage, 72 BMD-related pleiotropic SNPs were identified, and 12 SNPs located in 11 loci on 8 chromosomes were replicated in the UK Biobank. These SNPs were associated, in addition to BMD, with 14 different phenotypes. Most pleiotropic associations were exhibited by rs479844 (*AP5B1, OVOL1* genes), which was associated with dermatological and allergic diseases, and rs4072037 (*MUC1* gene), which was associated with magnesium levels and gastroenterological cancer. In conclusion, 12 BMD-related genome-wide significant SNPs showed pleiotropy with nonbone phenotypes. Pleiotropic associations can deepen the genetic understanding of bone-related diseases by identifying shared biological mechanisms with other diseases or traits.

## Introduction

Osteoporosis is the most common metabolic bone disorder globally, causing low trauma fractures that lead to significant morbidity and mortality. Especially in high-income countries, osteoporotic fractures lead to a substantial loss of healthy life-years in older adults.^1^ Osteoporosis is characterized by impaired bone quality and/or reduced bone mass, resulting from an imbalance between bone formation and resorption. Noninvasive diagnosis of osteoporosis currently relies heavily on measurement of bοne mineral density (BMD) by dual energy X-ray absorptiometry (DXA), which is the most widely used predictor of osteoporotic fractures.^2^

Genetic factors play a major role in the pathogenesis of osteoporosis, as reflected by the high heritability of many components of bone strength.^3^ Various genetic studies have contributed to a major breakthrough in unraveling new genetic associations of osteoporosis by identifying more than 500 loci in the last 10 years.^4–6^ Despite impressive progress in the field, a considerable area of osteoporosis-related heritability remains uncharted. This is partly due to parameters related to the disease itself, e.g., phenotypic variability (endophenotypes—“disease within the disease”)^7^ also including potentially existing phenocopies but also due to the use of the stringent *P* value < 5 × 10^−8^ genome-wide significance threshold that makes it difficult to identify many “true” genetic markers with small genetic risks that contribute to the overall phenotype even with underlying very large sample sizes.^8^ Various approaches have been proposed to complement the identification of valid genetic markers using genome-wide analyses, including tests for pleiotropy and other pathway analyses.^9,10^

Genetic pleiotropy occurs when a genetic variant (e.g., a single nucleotide polymorphism (SNP) is associated independently with two or more distinct phenotypes (diseases or quantitative traits). Systematic appraisals of the pertinent, rapidly accumulating epidemiological evidence suggest a prominent presence of genetic markers with a pleiotropic effect; the number of associating phenotypes per gene reported in the NHGRI-EBI Catalog ranges from 1 to 53, with 44% of genes associated with more than one phenotype.^11^ Various approaches have been used to investigate the potential shared genetic basis for different phenotypes, and robust evidence of pleiotropy has already accumulated for distinct disease entities, such as Parkinson’s disease and autoimmune diseases or body mass index and coronary artery disease.^12,13^

An important initial step in the process of revealing pleiotropic loci associated with complex phenotypes is to examine SNPs that have already been independently associated with one or more different phenotypes using the statistically stringent genome-wide association studies (GWAS) framework. Such an approach could reveal possible common pathways through which these polymorphisms act and therefore provide a contribution to the field of prevention, diagnosis, therapy, and prognosis or identify a simple correlation without pathophysiological background. The aim of the present study is to identify pleiotropic genetic variants associated with BMD and to better describe the shared genetic determinants between BMD and distinctive nonbone phenotypes, which would lay the foundations for further understanding of the potential relationships underlying BMD and these phenotypes.

## Results

### Selection of nonbone-related genetic loci and bone pleiotropy discovery phase

**Fig. 1:**
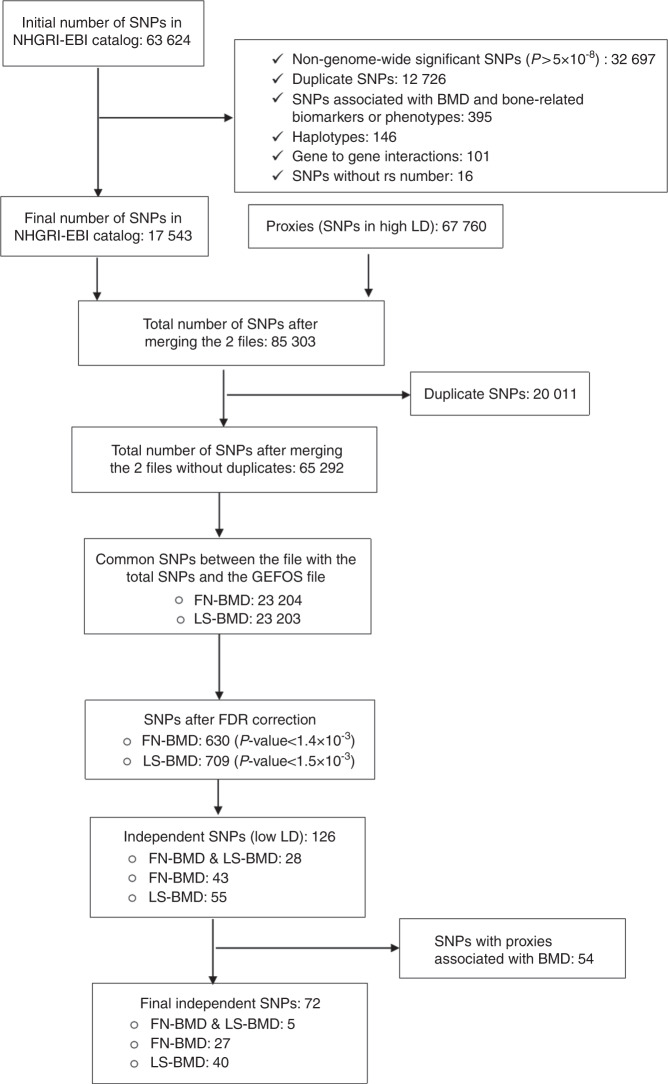
Flow diagram explaining the process for the selection of nonbone-related genetic loci in the bone pleiotropy discovery phase. BMD bone mineral density, FDR false discovery rate, FN femoral neck, GEFOS Genetic Factors for Osteoporosis, LD linkage disequilibrium, LS lumbar spine, NHGRI-EBI National Human Genome Research Institute-European Bioinformatics Institute, SNP single nucleotide polymorphism

As of December 14, 2017, the NHGRI-EBI Catalog included a total of 63 624 SNP-phenotype associations, of which 17 543 SNPs and their 67 760 proxies were selected for further analysis (Fig. 1). For the BMD traits, after removing the duplicate SNPs, we ended up with the 23 204 and 23 203 common variants present in the Genetic Factors for Osteoporosis (GEFOS) Consortium for an association with femoral neck (FN) BMD and lumbar spine (LS) BMD, respectively. The threshold for significance based on the false discovery rate (FDR) was 1.4 × 10^−3^ for FN-BMD, yielding a total of 630 SNPs, while for LS-BMD, the *P* value threshold was set at 1.5 × 10^−3^, yielding a total of 709 SNPs. Of these, we identified 126 independent SNPs for BMD, and 54 SNPs were further dropped since they were proxies to SNPs that have previously been associated with BMD, giving a final number of 72 independent pleiotropic SNPs associated with BMD (Supplementary Table 1).

The SNP exhibiting the greatest number of pleiotropic associations was rs3184504, which in addition to BMD was also associated with 22 different nonbone phenotypes, as well as SNPs rs479844, rs6675401 and rs2735343, which were associated with three nonbone phenotypes. The 72 independent SNPs were located in 64 loci on 20 different chromosomes; 53 of them were indexed in the NHGRI-EBI Catalog, whereas 19 SNPs were proxies of SNPs in the NHGRI-EBI Catalog that were not previously associated with BMD. The most common loci were 1p31.3 (*n* = 2), 2q33.1 (*n* = 2), 3p21.31 (*n* = 2), 4p16.3 (*n* = 2), 5q14.3 (*n* = 2), 6p21.32 (*n* = 2), 12p12.1 (*n* = 2), and 18q21.33 (*n* = 2). There were 90 different associated phenotypes with the most common being blood protein levels (*n* = 5 SNPs), educational attainment (years of education) (*n* = 4 SNPs), height (*n* = 4 SNPs), waist circumference (*n* = 3 SNPs), and inflammatory bowel disease (*n* = 3 SNPs). The discovery samples assessed various ancestral groups, the most common being European only (74.38%), Japanese only (3.31%), and Han Chinese only (3.31%). The minor allele frequency of the genetic marker under study also varied considerably with the mean, standard deviation, minimum, and maximum at 0.31, 0.13, 0.04, and 0.50, respectively.

### Bone pleiotropy replication in UK Biobank

In our two-stage design, we attempted replication of the 72 independent SNPs using UK Biobank as the replication resource. Analysis was performed for the readily available calculated heel BMD variable, as well as for the recalculated heel estimated BMD (eBMD) in unrelated UK Biobank participants of European descent. Demographic characteristics for the analyzed UK Biobank subjects are provided in Table 1. We identified 12 genome-wide significant pleiotropic SNPs located in 11 loci on 8 chromosomes (Table 2, Supplementary Table 2). Nine SNPs were reported in the NHGRI-EBI Catalog, whereas three SNPs were proxies (Supplementary Table 3).

**Table 1:** Demographic characteristics of the analyzed UK Biobank participants

Demographic characteristics	Total population (*N* = 417 629)	Females (*N* = 228 236, 54.65%)	Males (*N* = 189 393, 45.35%)
	Mean (SD, Min–Max)	Mean (SD, Min–Max)	Mean (SD, Min–Max)
Age at recruitment/years	56.76 (7.98, 38–73)	56.57 (7.89, 39–71)	57.00 (8.07, 38–73)
Weight/kg	77.93 (15.69, 30–197.7)	71.35 (13.89, 30–196)	85.87 (13.97, 40.8–197.7)
Left heel eBMD*/Z*-score	−0.001 9 (0.996 3, −3.894 4 –3.998 0)	−0.003 1 (0.993 7, −3.894 4 –3.998 0)	−0.000 3 (0.999 4, −3.417 6 –3.989 6)

*eBMD* estimated bone mineral density, *N* number of participants, *SD* standard deviation, *Min* minimum, *Max* maximum

**Table 2:** Pleiotropic genome-wide significant SNPs associated with heel BMD *Z*-score and nonbone phenotypes in the replication phase in the UK Biobank

SNP	Locus	Gene in this region	Associated phenotype(s) in NHGRI-EBI catalog	Direction of associated phenotype	Direction of BMD
rs3118905	13q14.3	*DLEU1*	Height	↑	↓
rs4963975	12p12.1	*SSPN*	Waist circumference	↓	↓
rs6599389	4p16.3	*TMEM175*	Parkinson’s disease	↑	↓
rs884127	1q41	*SLC30A10*	Urinary electrolytes (magnesium/calcium ratio)	↑	↓
rs9668810^a^	12p12.1	*SSPN*	Male-pattern baldness	↓	↓
rs479844	11q13.1	Intergenic^d^	Atopic dermatitis	↓	↓
rs479844	11q13.1	Intergenic^d^	Atopic march	↓	↓
rs479844	11q13.1	Intergenic^d^	Allergic disease (asthma, hay fever or eczema)	↓	↓
rs7221743^b^	17q11.2	*EFCAB5*	Coffee consumption	↑	↓
rs301800	1p36.23	*RERE*	Educational attainment	↓	↓
rs7899547	10q21.1	Intergenic^e^	Blood protein levels	NR	↓
rs9303601	17q21.31	*AC004596.1*	High light scatter reticulocyte red cells	↓	↓
rs4072037	1q22	*MUC1*	Magnesium levels	↑	↓
rs4072037	1q22	*MUC1*	Noncardia gastric cancer	↑	↓
rs4776908^c^	15q23	*AAGAB*	Waist circumference	↑	↓

*BMD* bone mineral density, *NR* not reported, *SNP* single nucleotide polymorphism

^a^Proxy SNP for the variant rs9668810 is rs9300169

^b^Proxy SNP for the variant rs7221743 is rs9902453

^c^Proxy SNP for the variant rs4776908 is rs7166081

^d^Nearest genes for the intergenic variant rs479844 are *AP5B1* and *OVOL1*

^e^Nearest gene for the intergenic variant rs7899547 is *MBL2*

Seeking to ensure that these 12 markers correspond to lead SNPs for both involved phenotypes, we found that 9 out of the 12 potential pleiotropic SNPs identified in our study are in LD (*r*^2^ = 0.11–0.99) with the signals described by Morris et al.^6^, the most recent and comprehensive effort on eBMD and fracture (Supplementary Table 9). LD plots for these SNP pairs are provided in Supplementary Figs. 1–8. In our effort, we identified three additional signals (rs6599389, rs9303601, and rs4776908) that show *r*^2^ < = 0.1 with the signals identified in the Morris et al. effort.^6^ However, all three signals fall within a 500 kb distance from a lead “Morris” SNP. Moreover, these three signals were genome-wide significant for the nonbone phenotypes, passed the FDR threshold for BMD and were genome-wide significant in the eBMD replication effort in the UK Biobank.

**Fig. 2:**
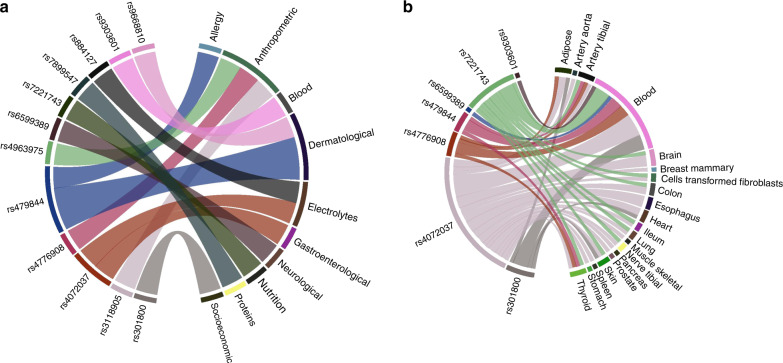
Schematic representation of the SNP-phenotype associations based on (**a**) the replication analysis in UK Biobank and (**b**) the bioinformatics results derived from PhenoScanner analysis

These 12 SNPs were associated with 14 different phenotypes classified into 10 different groups (Fig. 2). The variants rs479844 and rs4072037 exhibited the most pleiotropic associations, which in addition to BMD were also associated with 3 and 2 different nonbone phenotypes. The other pleiotropic SNPs were associated with BMD and one extra phenotype. Sensitivity analysis based on eBMD yielded similar results (Supplementary Table 2).

In addition, the effect of different ancestries in the NHGRI-EBI Catalog discovery phase was tested. Specifically, we compared the effect estimates for each pleiotropic SNP associated with the same phenotype in different ancestries, and there did not seem to be any difference (Supplementary Table 4). Relevant data were available for 3 out of the 12 pleiotropic SNPs. Moreover, the direction of effect for the majority of the replicated SNPs (9 out of 12 SNPs, 75%) was the same between GEFOS and UK Biobank (Supplementary Table 5).

### Bioinformatics analysis

We chose to pursue further in silico assessment for the 12 genome-wide significant pleiotropic SNPs. Using PhenoScanner in the expression quantitative trait loci (eQTL) option, we demonstrated that 7 out of the 12 SNPs were significantly associated with gene expression in multiple human nonbone tissues and cells at a genome-wide significant level, including adipose, artery aorta, artery tibial, brain, breast mammary, cells transformed fibroblasts, colon, esophagus, heart, ileum, lung, skeletal muscle, tibial nerve, pancreas, prostate, skin, spleen, stomach, thyroid, whole and peripheral blood (Supplementary Table 6, Fig. 2). In addition, bioinformatics analysis revealed that 4 out of these 7 pleiotropic SNPs produced gene expression levels in nonbone tissues different from the ones expected from their association in the NHGRI-EBI Catalog (Supplementary Table 7). Further analysis using PhenoScanner in the metabolites option showed that none of the 12 pleiotropic SNPs was associated with any metabolite at a genome-wide significant level.

### Genes, gene expression and phenotype data consolidation

The identified phenotypes that were associated with BMD loci varied in terms of their characteristics and genetic background (Table 2, Supplementary Table 3). Notably, the observed genetic variant associated with two or more phenotypes in our study might be the true causal variant, but it might also be in strong linkage disequilibrium (LD) with the causal variant. Similarly, the reported gene does not mean that it is the causal gene. It actually represents the closest gene to each pleiotropic SNP.

### Parkinson’s disease

Our study indicated that the variant rs6599389 in the *TMEM175* gene (effect allele A) was associated with a decrease in BMD and an increase in Parkinson’s disease risk. *TMEM175* is a protein-coding gene that is responsible for potassium conductance in endosomes and lysosomes. eQTL data showed that this signal was associated with gene expression in blood and peripheral blood. Our finding is in line with available epidemiological evidence.^14,15^ There are a number of potential mechanisms that could lead to reduced BMD in patients with Parkinson’s disease, in addition to a common biological pathway, such as weight loss, malnutrition, vitamin D deficiency, reduced exposure to sunlight, immobility, reduced muscle strength, and the use of specific antiparkinsonian drugs, particularly levodopa, which can cause hyperhomocysteinemia.

### Noncardia gastric cancer

Our study also revealed that the variant rs4072037 located in the *MUC1* gene (effect allele T) was associated with a decrease in BMD and an increase in the risk of noncardia gastric cancer. *MUC1* encodes a membrane-bound protein that is a member of the mucin family. Mucins play an essential role in forming protective mucous barriers on epithelial surfaces. Overexpression, aberrant intracellular localization and changes in glycosylation of these proteins have been associated with carcinomas. eQTL lookup for this signal revealed gene expression in the stomach, as well as in thyroid, lung, pancreas, breast mammary tissue, prostate, spleen, muscle skeletal, skin, blood, cells EBV-transformed lymphocytes, lymphoblastoid cell lines, peripheral blood monocytes, artery aorta, artery tibial, heart left ventricle, heart atrial appendage, esophagus mucosa, esophagus muscularis, esophagus gastroesophageal junction, normal prepouch ileum, colon transverse, colon sigmoid, cells transformed fibroblasts, adipose subcutaneous tissue, adipose visceral omentum, nerve tibial, brain cortex, brain cerebellar hemisphere, and brain cerebellum.

Epidemiological evidence has shown that bone disorders are observed in gastric cancer patients and specifically reduce bone mass, osteoporosis and/or associated fractures.^16^ However, older age, female sex, malabsorption, malnutrition, low body weight, cancer itself and its treatment (e.g., chemotherapy, gastrectomy) are independent risk factors for osteoporosis in gastric cancer patients.

### Dermatological and allergic diseases

We also identified that the rs479844 variant located near the *AP5B1* and *OVOL1* genes (effect allele A) was associated with a decrease in BMD and a decrease in the risk of atopic dermatitis, atopic march (atopic dermatitis in infancy and subsequent allergic rhinitis and asthma in later childhood)^17^ and allergic disease (asthma, hay fever or eczema). *AP5B1* is a protein-coding gene that, as part of the AP-5 complex (fifth adaptor protein), may be involved in endosomal transport. *OVOL1* is a protein-coding gene and a putative transcription factor. Notably, the *AP5B1* gene is associated with osteogenesis imperfecta type XII, which is an autosomal recessive form characterized by generalized osteoporosis, mild bone deformations, recurrent fractures, delayed teeth eruption, and white sclerae. eQTL analysis showed that this variant was associated with gene expression in peripheral blood, lymphoblastoid cell lines, EBV-transformed lymphocytes, thyroid, and artery tibia. Epidemiological studies have shown that atopic dermatitis, asthma, and eczema are significantly associated with low BMD due to various factors, e.g., corticosteroid use, diets that avoid milk, and other essential foods resulting in insufficient calcium intake, less physical activity, vitamin D deficiency, and the inflammatory nature of the diseases.^18–21^

In addition, the signal rs9668810 located in the *SSPN* gene (effect allele T) was associated with a decrease in BMD and a decrease in male-pattern baldness risk. eQTL analysis for this signal showed that it was not associated with gene expression in any human nonbone tissue at a genome-wide significant level. Epidemiological evidence indicates a possible common genetic background between BMD and male-pattern baldness,^22^ and experimental data suggest that neural crest-derived hair follicle cells possess a capacity for osteoblastic differentiation.^23^

### Anthropometric measurements

We observed one pleiotropic association with height. The rs3118905 signal located in the *DLEU1* gene (effect allele G) was associated with a decrease in BMD and an increase in height. *DLEU1* is a nonprotein-coding RNA gene that was originally identified as a potential tumor suppressor gene. Based on the eQTL lookup, this variant was not associated with gene expression in any human nonbone tissue. Epidemiologically, there is accumulated evidence indicating a common genetic background between BMD and height,^24^ and height is considered a clinical risk factor for fracture.

We identified another two independent BMD signals associated with waist circumference. The variant rs4776908 at the 15q23 locus in the *AAGAB* gene (effect allele T) was associated with a decrease in BMD and an increase in waist circumference, while rs4963975 at the 12p12.1 locus in the *SSPN* gene (effect allele G) was associated with a decrease in BMD and a decrease in waist circumference, which brings in question the direction of pleiotropy at the SNP level. *AAGAB* encodes a protein involved in clathrin-coated vesicle trafficking and may also be involved in endocytic recycling of growth factor receptors such as EGFR. *SSPN* encodes a member of the dystrophin-glycoprotein complex that spans the sarcolemma and provides a structural link between the subsarcolemmal cytoskeleton and the extracellular matrix of muscle cells. eQTL analysis for the variant rs4776908 indicated gene expression in subcutaneous adipose tissue, as well as in artery tibia, thyroid, and blood. However, eQTL analysis for the signal rs4963975 revealed that it was not associated with gene expression in any human tissue. The results of previous investigations of the association between obesity and bone health have been inconsistent.^25–27^ Obesity is traditionally considered to be beneficial to bone metabolism due to the positive effect of mechanical loading conferred by body weight on bone formation. However, accumulating data suggest that obesity is detrimental to bone health mainly due to upregulated proinflammatory cytokine production and, as a result, decreased osteoblast differentiation and increased osteoclast activity.

### Blood and urinary electrolyte levels

We identified that the variant rs4072037 located in the *MUC1* gene (effect allele T) was associated with an increase in BMD and a decrease in magnesium levels. As described before, *MUC1* encodes a membrane-bound protein that is a member of the mucin family. Mucins play an essential role in intracellular signaling, as well as in forming protective mucous barriers on epithelial surfaces. As mentioned before, eQTL analysis showed that this variant was associated with gene expression in various human nonbone tissues. Magnesium deficiency has been linked mainly to low BMD in observational and animal studies through direct action on crystal formation and bone cells, as well as indirectly by impacting the secretion and action of parathyroid hormone and promoting low-grade inflammation.^28^ However, our findings are in line with the higher BMD observed in patients with low plasma magnesium levels as a result of Gitelman’s syndrome.^29^ Pathophysiologically, bone anabolic effects, and inhibition of bone resorption are observed in severe magnesium deficiency through, in part, a defect in secretion and/or skeletal responsiveness to parathyroid hormone and vitamin D metabolites.^30^

In addition, the variant rs884127 located in the *SLC30A10* gene (effect allele A) was associated with a decrease in BMD and an increase in the urinary magnesium/calcium ratio. *SLC30A10* is a protein-coding gene that plays a pivotal role in manganese transport, maintaining manganese levels and conferring protection against manganese-induced cell death. It also mediates zinc transport into endosomes, preventing zinc toxicity. eQTL lookup showed that this variant was not associated with gene expression in any human nonbone tissue. There is available epidemiological evidence connecting urinary calcium concentration with bone health. Specifically, hypercalciuria is frequently present in a significant percentage of patients with BMD loss, which is thought to be the result of high bone turnover with excessive bone resorption.^31,32^

### Blood protein levels

The variant rs7899547 located near the *MBL2* gene (effect allele T) was associated with BMD and levels of mannose-binding protein C, which is a calcium-dependent lectin involved in innate immune defense. *MBL2* encodes a protein that is secreted by the liver as part of the acute-phase response and is involved in innate immune defense. eQTL analysis for this signal showed that it was not associated with gene expression in any human nonbone tissue. Epidemiological data indicate multiple genetic correlations between blood proteins and BMD^33^ and that proteins are an essential factor for bone health.^34^

### Red blood cells

The variant rs9303601 located in the *AC004596.1* gene (effect allele A) was associated with a decrease in BMD and a decrease in high light scatter reticulocyte red cells. *AC004596.1* is a nonprotein-coding RNA gene, and its novel transcript is antisense to *ATXN7L3*, which is a protein-coding gene. eQTL analysis showed that this signal was associated with gene expression in blood. Epidemiological evidence has indicated that hematological diseases accompanied by chronic anemia (e.g., beta thalassemia major, sickle cell anemia, and chronic hemolytic anemia) are characterized by the concomitant development of osteoporosis given the interconnections of bone and hematopoietic cells.^35,36^

### Education attainment

Our study indicated that the variant rs301800 located in the *RERE* gene (effect allele C) was associated with a decrease in BMD and a decrease in years of education. *RERE* is a protein-coding gene that has a role in transcriptional repressor during development, as well as in the control of cell survival. eQTL analysis revealed that this signal was associated with gene expression in blood, peripheral blood monocytes, left ventricle of the heart, adipose visceral omentum, and skin. Epidemiological evidence has shown that women with higher education have a higher probability of undergoing DXA scans and closely observe any changes in BMD,^37^ and subjects with lower education levels are more likely to have lower BMD.^38^

### LD score regression

The LD score regression analyses yielded 60 genetic correlation estimates between LS-BMD or FN-BMD and different diseases/traits that were already replicated in the UK Biobank through our approach. We identified 14 nominally significant correlations between BMD and our pleiotropic replicated phenotypes. Specifically, the estimated genetic correlation between BMD and height, blood protein levels (albumin) and hair/balding pattern 3 (moderate hair loss) or 4 (severe hair loss) was significant (*P* value < 0.05) and positive. The genetic correlation between BMD and hay fever or allergic rhinitis was also nominally significant but negative. The point estimates ranged from −0.162 8 to 0.388 5. In addition, the genetic correlations between BMD and the other pleiotropic phenotypes found in our study (asthma, dermatitis, eczema, Parkinson’s disease, waist circumference, and age completed full-time education) were not significant. Supplementary Table 8 summarizes the available genetic correlation results.

## Discussion

Using a genome-wide pleiotropy scan and a two-stage design, we examined more than 63 500 SNPs previously associated with various nonbone phenotypes looking for association with BMD in more than 80 000 individuals of the GEFOS Consortium and replicated the strongest SNPs in an independent sample of more than 400 000 subjects of European ancestry in the UK Biobank. Twelve genome-wide significant SNPs were found to be associated with both BMD and other nonbone phenotypes. The identified phenotypes varied greatly based on their genetic background and clinical characteristics, and many observed associations were in line with the eQTL analysis and the available epidemiological data. Moreover, LD score regression was significant for the genetic correlation between BMD and height, male-pattern baldness, blood protein levels (albumin), hay fever, and allergic rhinitis.

Pleiotropic effects of GWAS-discovered SNPs have been identified in the past, and different types of pleiotropy have been described.^39^ Biological pleiotropy occurs when a single causal variant is related to multiple phenotypes, mediated pleiotropy occurs when a variant is related to one phenotype that is, itself, causally related to a second phenotype, and spurious pleiotropy includes various sources of bias. Biological and mediated pleiotropy can provide useful data regarding disease pathophysiology and biological mechanisms underlying seemingly unrelated phenotypes. While we were not able within this effort to distinguish whether the identified SNPs in our study act through biological or mediated pleiotropy owing to data limitations, we believe that the possibility of observing spurious pleiotropic associations is unlikely due to the use of stringent analysis and large sample sizes and acknowledge that a structured functional lookup would be informative.

In previous efforts, GWAS have revealed a number of SNPs that are associated in a pleiotropic way with BMD and apparently unrelated non-BMD diseases and traits.^40–42^ In parallel to our work, Morris et al.^6^ undertook a comprehensive assessment of human and murine genetic determinants of eBMD from the heel and identified a total of 518 genome-wide significant loci (301 novel), explaining 20% of the total variance in BMD. In the current study, designed and implemented before the UK Biobank eBMD data were analyzed using a GWAS framework, we followed a different statistical methodology based on the pleiotropy context, trying to identify and critically characterize BMD-related pleiotropic variants. The identified signals using our approach show considerable overlap with the abovementioned GWAS effort, reflecting the usefulness of the process we implemented and its potential value when no additional GWAS data are available.

Based on our study results, a decrease in BMD is associated with an increase in the risk of Parkinson’s disease and noncardia gastric cancer, as well as a decrease in the risk of male-pattern baldness, atopic dermatitis, and allergic disease. In addition, a decrease in BMD is associated with an increase in height, urinary magnesium/calcium ratio, and blood magnesium levels, as well as a decrease in high light scatter reticulocyte red cells and lower education level. However, it is important to note that these associations are only hypothesis-generating. They must be further validated keeping in mind the various complicated interactions between different genes, as well as the effect of the environment itself.

In accordance with GWAS experiments and based on the pleiotropy phenomenon, in this study, we found that there are specific pleiotropic markers for BMD and different nonbone phenotypes. Therefore, using alternative methodological approaches, we should be able to identify the pleiotropic associations of various diseases and traits and deepen their genetic understanding. The high percentage of the SNPs that survived the replication phase may be an indication of the highly pleiotropic nature of the human genomic associations for musculoskeletal traits, as has been described before.^43^ In addition, in silico assessment showed that half of the 12 pleiotropic SNPs were significantly associated with gene expression in multiple nonbone tissues. Finally, one-third of the 12 SNPs drove gene expression in nonbone tissues different from the ones expected from their association in the NHGRI-EBI Catalog.

An important strength of this study is the fact that data from the large international collaboration GEFOS were used and replication analysis was performed in the large health resource UK Biobank. Indeed, using datasets with large sample sizes has proven to be an effective way to strengthen the genetic understanding of complex phenotypes.^44^ In addition, the less conservative method of FDR was used to avoid false negative findings, and proxies of the SNPs from the NHGRI-EBI Catalog were found to be more inclusive and avoid missing possible highly associated SNPs.

However, our approach is not without limitations. First, the analysis was restricted to variants included in the NHGRI-EBI Catalog; thus, variants identified in large candidate gene studies may have been missed. In addition, the strict threshold of *P* value 5 × 10^−8^ was used, and as a result, SNPs with weaker associations may not have been identified. GWAS focused on the common variants, and thus, the lowest minor allele frequency in our analysis was 0.04. We also used ultrasound data from the UK Biobank to examine bone mass associations instead of using the more conventional method of DXA. Moreover, we performed the initial search in the NHGRI-EBI Catalog in the discovery phase before the analysis, and the critical appraisal of the UK Biobank data and a formal colocalization assessment was not possible due to data limitations. Finally, possible sample overlap may exist between GEFOS data and relevant GWAS included in the NHGRI-EBI Catalog.

In conclusion, our findings suggest 12 pleiotropic BMD variants, as well as possible common genetic relationships with different nonbone phenotypes. Such a pleiotropic approach can be used to identify pleiotropic genetic variants and shared biologic pathways between seemingly unrelated phenotypes. Validation of these signals is needed by using additional biological and mechanistic data. This will not only deepen our understanding of the genetic architecture and the missing heritability of complex human phenotypes but will also have clinically important implications in the field of prevention, diagnosis, therapy, and prognosis.

## Materials and methods

### Selection of nonbone-related genetic loci

We first sought to identify established genetic markers across the whole range of phenotypes. The NHGRI-EBI Catalog (a curated collection of all published GWAS) was searched with the latest update performed on December 14, 2017. The following SNPs were excluded: nongenome-wide significant SNPs (*P* value > 5 × 10^−8^), SNPs pertaining to gene–gene interactions, SNPs that were assessed within haplotypes, duplicate SNP entries, SNPs with missing rs numbers that could not be found using the data reported in the original GWAS, SNPs that have already been associated with BMD,^4,5^ and other SNPs associated with bone-related biomarkers and phenotypes (e.g., osteoporosis, fracture, and calcium levels). We defined pleiotropy according to an established and most commonly used set of criteria: 1) the associated phenotypes are not pathophysiological counterparts, 2) one phenotype is not a subset of the other, 3) one phenotype is not used to calculate the other, 4) the phenotypes are not similar or strongly correlated such that they might not be measures of the same genetic effect, and 5) one phenotype is not a known causal factor for the other.^45^

The software tool SNiPA^46^ was used to identify the “perfect proxies” of the abovementioned SNPs (SNPs in high LD, *r*^2^ = 1). By merging the initially identified SNPs with the retrieved proxies and removing the duplicate SNPs, we created a list of genome-wide significant SNPs and their proxies across all disease entities and traits.

### Bone pleiotropy discovery phase

We then identified which of these markers were pleiotropically associated with BMD within the large international collaboration GEFOS in more than 80 000 subjects using FN-BMD and LS-BMD as phenotypes.^4^ Statistical models used in the GEFOS analyses were adjusted for age,^2^ weight, and principal components to account for population stratification. We focused on FN-BMD and LS-BMD because osteoporotic fractures at the hip and spine are the most common and serious fracture types that have been intensively studied in genetic and epidemiological studies related to osteoporosis.^47^ For the selection of putative BMD-related pleiotropic markers, we used a threshold that accounted for multiple testing, implementing the less conservative technique of FDR at 5% by applying the Simes procedure, which is closely related to the Benjamini–Hochberg correction^48,49^ instead of a Bonferroni correction. Using the LDlink tool,^50^ we identified the independent SNPs for FN-BMD and LS-BMD (SNPs in low LD, *r*^2^ < 0.2). SNPs that had proxies already associated with BMD at a GWAS level in SNiPA or in previously published work^4,5^ were removed.

### Bone pleiotropy replication in UK Biobank

We then sought to identify whether these putative genetic markers would be replicated at the globally accepted genome-wide significance level (*P* value < 5 × 10^−8^) to avoid false positive results. The large cohort of UK Biobank participants of European ancestry with available left heel bone ultrasound and genomic data was used. The UK Biobank is a large, population-based cohort study in subjects aged 40–69 years across the UK.^51^ The full release of data from UK Biobank contains genotypes of 488 377 participants. SNPs were imputed centrally by UK Biobank using a reference panel that merged the UK10K and 1 000 Genomes Phase 3 panel, as well as the Haplotype Reference Consortium panel.^52^ The UK Biobank obtained ethical approval from the NHS National Research Ethics Service (17th June 2011, Ref 11/NW/0382). All participants provided written informed consent to participate.

We focused on heel ultrasound measurements because DXA UK Biobank data were not performed anywhere near the numbers for heel ultrasound. Subjects with data available at baseline were included in the analysis. The heel ultrasound device measures the speed of sound (in meters/second) and broadband ultrasound attenuation (in decibels/megahertz), which are combined to provide the quantitative ultrasound index or stiffness. The device system software automatically calculates BMD (in g·cm^−2^) from the quantitative ultrasound index. UK Biobank uses Vox software to automatically collect data (direct entry), while manual entry is used whenever the direct entry method fails. For our analysis, we also considered manual entries whenever direct entries were not available.^53^ Subjects exceeding the following thresholds for speed of sound, broadband ultrasound attenuation, and calculated BMD were excluded as outliers: (male ≤1 450 and ≥1 750 m·s^−1^, female ≤1 455 and ≥1 700 m·s^−1^), (male ≤27 and ≥138 dB·MHz^−1^, female ≤22 and ≥138 dB·MHz^−1^), and (male ≤0.18 and ≥1.06 g·cm^−2^, female ≤0.12 and ≥1.025 g·cm^−2^), respectively. Using *KING* software, we identified related individuals by estimating kinship coefficients for all pairs of samples and recorded the pairs of degree 3 or closer (kinship coefficient ≥1/2^(9/2)^) (ref. ^52^) Then, we arbitrarily retained data from only one member of each pair. For each participant, we estimated the gender-specific standardized *Z*-score by running a linear regression on calculated BMD values adjusting for age, age^2^ and weight. We excluded *Z*-scores of the assessed phenotype lying outside values of −4 and 4 from our analyses, as done previously.^4^

Information for SNPs and related genes was derived from the genome browser Ensembl release 95 (ref. ^54^) and data for gene function were derived from the GeneCards database.^55^

### Statistical and bioinformatics analysis

For the SNPs that surpassed the significance level in the replication analysis, we performed a linear regression on the *Z*-score after adjustment for the first 15 principal components, and we obtained their beta coefficients combined with their 95% confidence intervals. In addition to the original UK Biobank variable for heel BMD, sensitivity analysis was also performed based on eBMD calculated from the following formula: eBMD = 0.002 592*(broadband ultrasound attenuation + speed of sound) − 3.687 (ref. ^56^). Due to potential inconsistencies with the UK Biobank file, variants rs7047907 and rs9268877, indexed in the NHGRI-EBI Catalog, were kept instead of their proxies rs7869321 and rs9269080, respectively, which had lower *P* values after implementing the FDR technique. Two investigators (M.A.C., G.M.) independently performed the association analysis in the replication stage. Statistical analysis was performed with the statistical packages Stata 12 (ref. ^57^) and PLINK 1.9 (ref. ^58^).

LD score regression was performed using the web-based tool LD Hub for all SNPs associated with FN-BMD and LS-BMD in the GEFOS Consortium to estimate the genetic correlation between BMD and different phenotypes derived from several published GWAS summary statistics included in LD Hub.^59^

PhenoScanner^60^ was examined for potential in silico functional impact of the pleiotropic SNPs resulting from the replication analysis and their proxies (*r*^2^ = 0.6). We reported eQTL—and metabolite—related data at a genome-wide significance level (*P* value < 5 × 10^−8^) to be more stringent and less inclusive.

## Supplementary information

Supplementary Material

Supplementary Figure 1

Supplementary Figure 2

Supplementary Figure 3

Supplementary Figure 4

Supplementary Figure 5

Supplementary Figure 6

Supplementary Figure 7

Supplementary Figure 8

Supplementary Tables

## Data Availability

Data from the GEFOS Consortium are available at http://www.gefos.org/?q=content/data-release-2012. Genetic data can be provided by the UK Biobank (approved application number 22102).
