# Supplementary material for: A genome-wide scan for pleiotropy between bone mineral density and nonbone phenotypes
Source: Bone Res. 2020 Jul 1;8:26. doi: 10.1038/s41413-020-0101-8 (PMC7329904; doi:10.1038/s41413-020-0101-8)
Supplement: Supplementary file 1 — Supplementary Material [file 41413_2020_101_MOESM1_ESM.docx]

**Supplementary Tables**

**Supplementary Table 1. Pleiotropic SNPs associated with FN/LS-BMD and non-bone phenotypes in the discovery phase.**

BMD, Bone Mineral Density; FN, Femoral Neck; GEFOS, Genetic Factors for Osteoporosis; LD, Linkage Disequilibrium; LS, Lumbar Spine; MAF, Minor Allele Frequency; NHGRI-EBI, National Human Genome Research Institute-European Bioinformatics Institute; NR, not reported; SE, standard error; SNP, Single Nucleotide Polymorphism.

^1^Refers to the available GEFOS phenotypes associated with a specific SNP; FN-BMD and LS-BMD, FN-BMD or LS-BMD. In case of both phenotypes being available, FN-BMD and LS-BMD, data for the phenotype with the smallest p-value were kept (marked in bold).

**Supplementary Table 2. Replication analysis for the left heel BMD Z-score of the original UK-Biobank variable and the estimated.**

A1, risk allele; eBMD, estimated Bone Mineral Density; L95, lower bound of 95% confidence interval; MAF, Minor Allele Frequency; nmiss, number of non-missing observations; SE, standard error; SNP, Single Nucleotide Polymorphism; U95, upper bound of 95% confidence interval.

**Supplementary Table 3. Characteristics of the pleiotropic genome-wide significant SNPs associated with heel BMD Z-score and non-bone phenotypes in the replication phase in UK Biobank.**

BMD, Bone Mineral Density; CI, Confidence Interval; EAF, Effect Allele Frequency; NA, not applicable; NR, not reported; OR, Odds Ratio; SNP, Single Nucleotide Polymorphism.

In case of multiple GWAS for the same SNP-phenotype association, the study with the largest sample size and the smallest p-value was kept.

**Supplementary Table 4. Effect of different ancestries in the NHGRI-EBI catalog.**

NA, not applicable; OR, odds ratio; SNP, Single Nucleotide Polymorphism.

**Supplementary Table 5. Direction of effect in GEFOS and UK Biobank for the 12 pleiotropic SNPs.**

A1, usually the minor allele in UK Biobank; SNP, Single Nucleotide Polymorphism.

**Supplementary Table 6. Bioinformatics analysis in PhenoScanner.**

Alleles, the alleles (aligned to the + strand) for the input SNP which correspond to the effect allele/other allele of the association alleles column; Association alleles, effect allele/other allele; Beta, association between the trait and the SNP expressed per additional copy of the effect allele; Description, the type of eQTL analyzed; dprime; the D' between the input SNP and the proxy SNP; EAF, effect allele frequency; Ensembl, the Ensembl ID for the nearest gene; Gene, the input gene; N, number of individuals; PMID, PubMed ID; pos_hg19, the hg19 position for the input SNP; Probe, the probe used to measure gene expression; Proxy alleles, the alleles (aligned to the + strand) for the proxy SNP which correspond to the effect allele/other allele of the association alleles column and are aligned according to the effect allele/other allele of the alleles column; Proxy indicator, a variable which equals 0 if the proxy SNP is the input SNP and 1 otherwise; proxy pos (hg19), the hg19 position for the proxy SNP; Proxy rsid, the rsID for the proxy SNP; r^2^, the *r*^2^ between the input SNP and the proxy SNP; rsid, the rsID for the input SNP; SNP, the input rsID or hg19 chromosome position; SE, standard error of beta; Source, the source of the data; Study, the name of the consortium/lead author of the study; Tissue, the tissue in which the eQTL were analysed; Year, the year the study was published.

**Supplementary Table 7. Pleiotropic SNPs and relevant phenotypes in NHGRI-EBI catalog and Phenoscanner.**

NA, not applicable; SNP, Single Nucleotide Polymorphism.

**Supplementary Table 8. Genetic correlation in LD Hub for the phenotypes that were replicated in our study.**

BMD, Bone Mineral Density; DVT, deep vein thrombosis; FN, Femoral Neck; gcov_int, cross-trait LD score regression intercept; gcov_int_SE, standard error of gcov_int; h2_int, single-trait LD score regression intercept for Trait 2; h2_int_SE, standard error of h2_int; h2_obs, observed scale h2 for Trait 2; h2_obs_SE, standard error of h2_obs; LS, Lumbar Spine; PMID, the PubMed ID of Trait 2; p-value, p-value for rg_h2_obs; rg, genetic correlation; SE, standard error of rg; Trait 1, uploaded trait; Trait 2, trait in LD Hub; UKBB, UK Biobank.

**Supplementary Table 9. Pairwise LD between the potential pleiotropic SNPs of our study and the SNPs reported by Morris *et al*.**

QRSID, Query SNP rsID (one of the potential pleiotropic SNPs of our study); RSID, Proxy SNP rsID (one of the SNPs reported by Morris *et al*.); RSALIAS, Proxy SNP alias rsID(s); CHR, Chromosome; POS1, Sentinel SNP Position; POS2, Proxy SNP Position; DIST, Distance; R2, LD r^2; D, LD D; DPRIME, LD D'; MAJOR, Proxy Allele A; MINOR, Proxy Allele B; MAF, Allele B Frequency; CMMB, Recombination Rate (CM/Mb); CM, Genetic distance.

**Supplementary Figures**

**Supplementary Figure 1.** LD plot for the SNP pair rs301800 - rs3765971.

**Supplementary Figure 2.** LD plot for the SNP pair rs479844 - rs10750766.

**Supplementary Figure 3.** LD plot for the SNP pair rs884127 - rs2647462.

**Supplementary Figure 4.** LD plot for the SNP pair rs3118905 - rs3118906.

**Supplementary Figure 5.** LD plot for the SNP pair rs4072037 - rs914615.

**Supplementary Figure 6.** LD plot for the SNP pair rs4963975 - rs10842704.

**Supplementary Figure 7.** LD plot for the SNP pair rs7899547 - rs12218358.

**Supplementary Figure 8.** LD plot for the SNP pair rs9668810 - rs7974900.

* We were not able to graphically confirm the presence of LD for the SNP pairs rs301800 – rs4908776 and rs7221743 – rs3760456 because the distance between them was larger than the default distance in the SNiPA plot.
