# Supplementary figures and images for: A genome-wide scan for pleiotropy between bone mineral density and nonbone phenotypes
Source: Bone Res. 2020 Jul 1;8:26. doi: 10.1038/s41413-020-0101-8 (PMC7329904; doi:10.1038/s41413-020-0101-8)

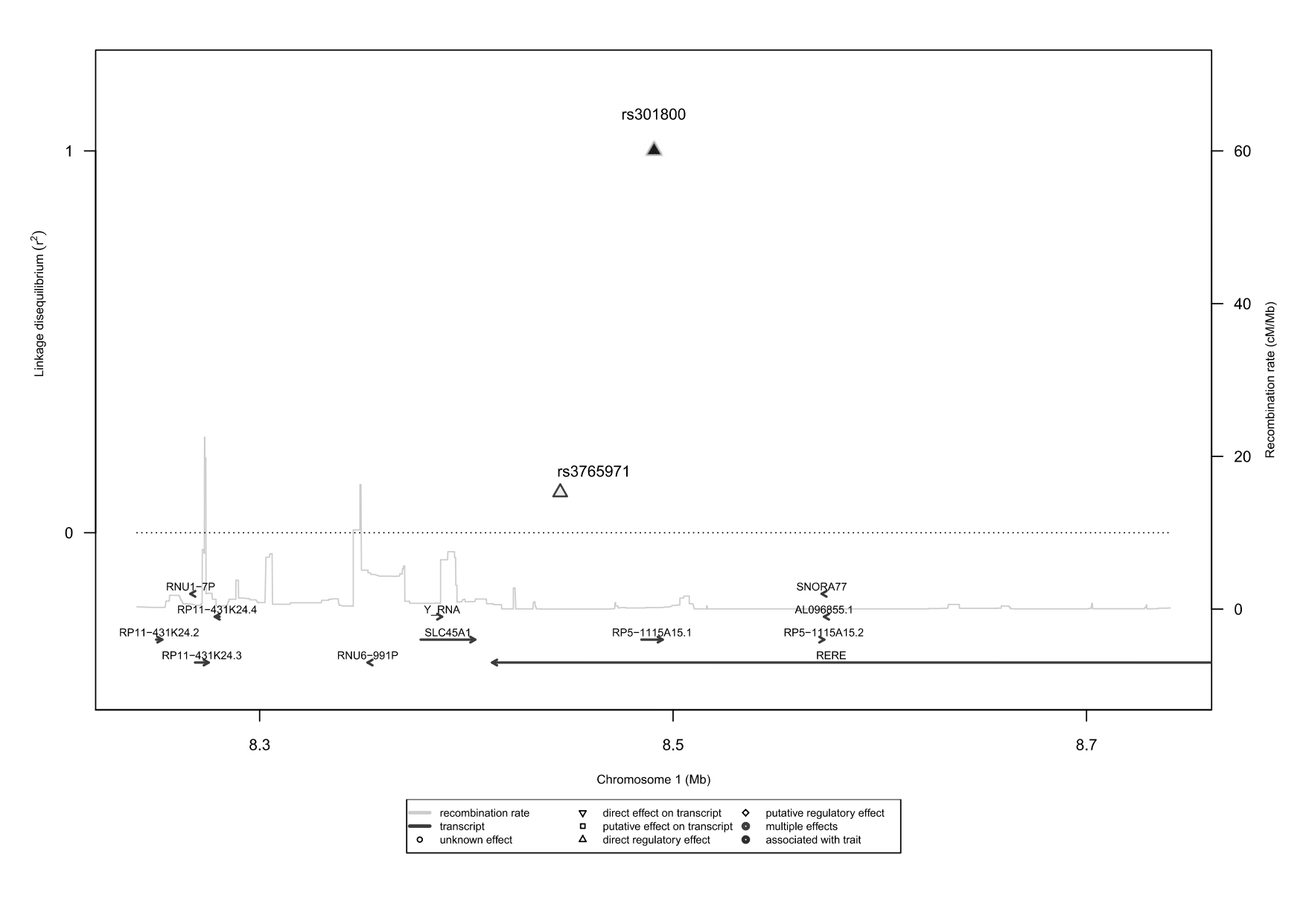

Supplement: Supplementary file 2 — Supplementary Figure 1 [file 41413_2020_101_MOESM2_ESM.jpg]

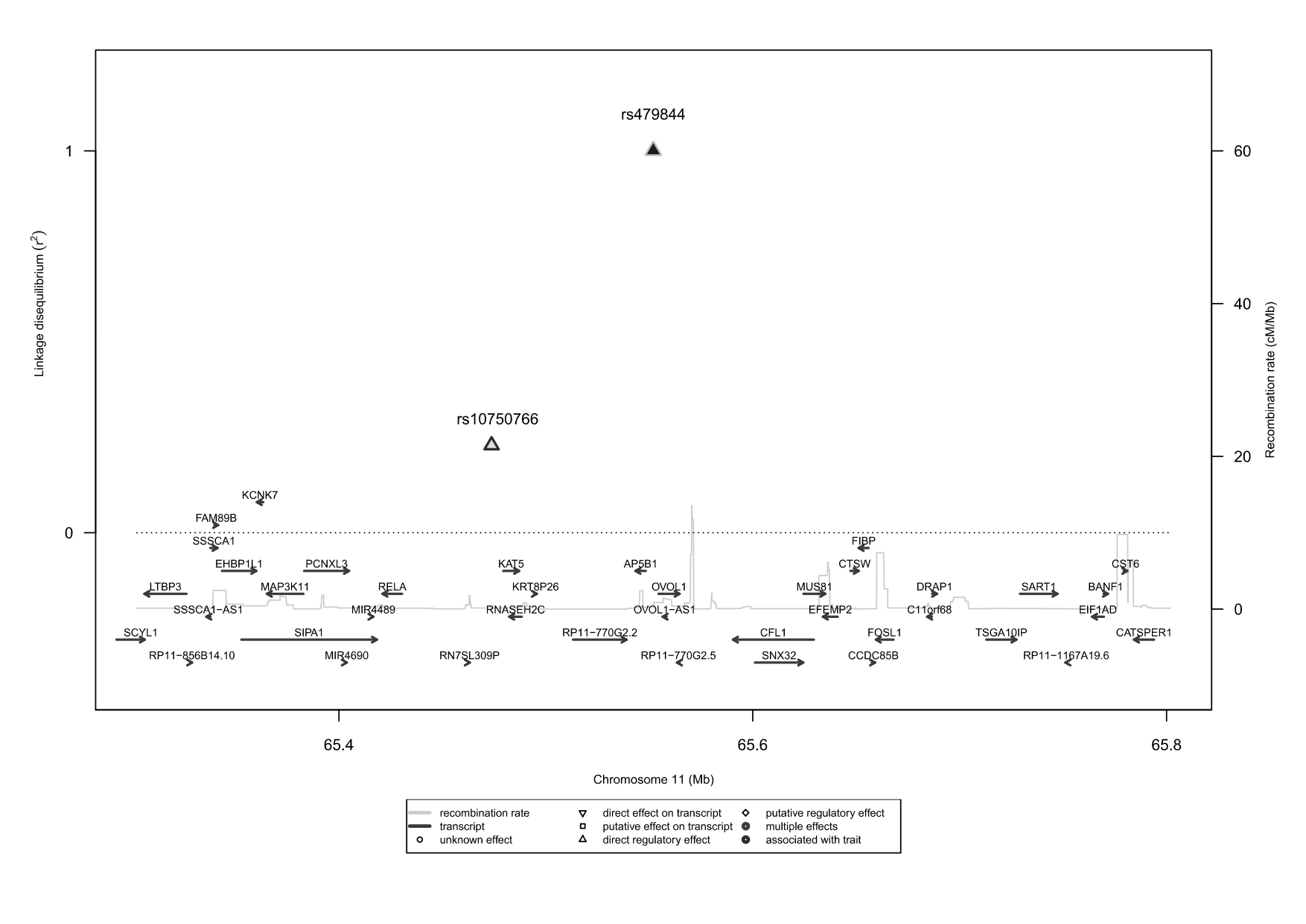

Supplement: Supplementary file 3 — Supplementary Figure 2 [file 41413_2020_101_MOESM3_ESM.jpg]

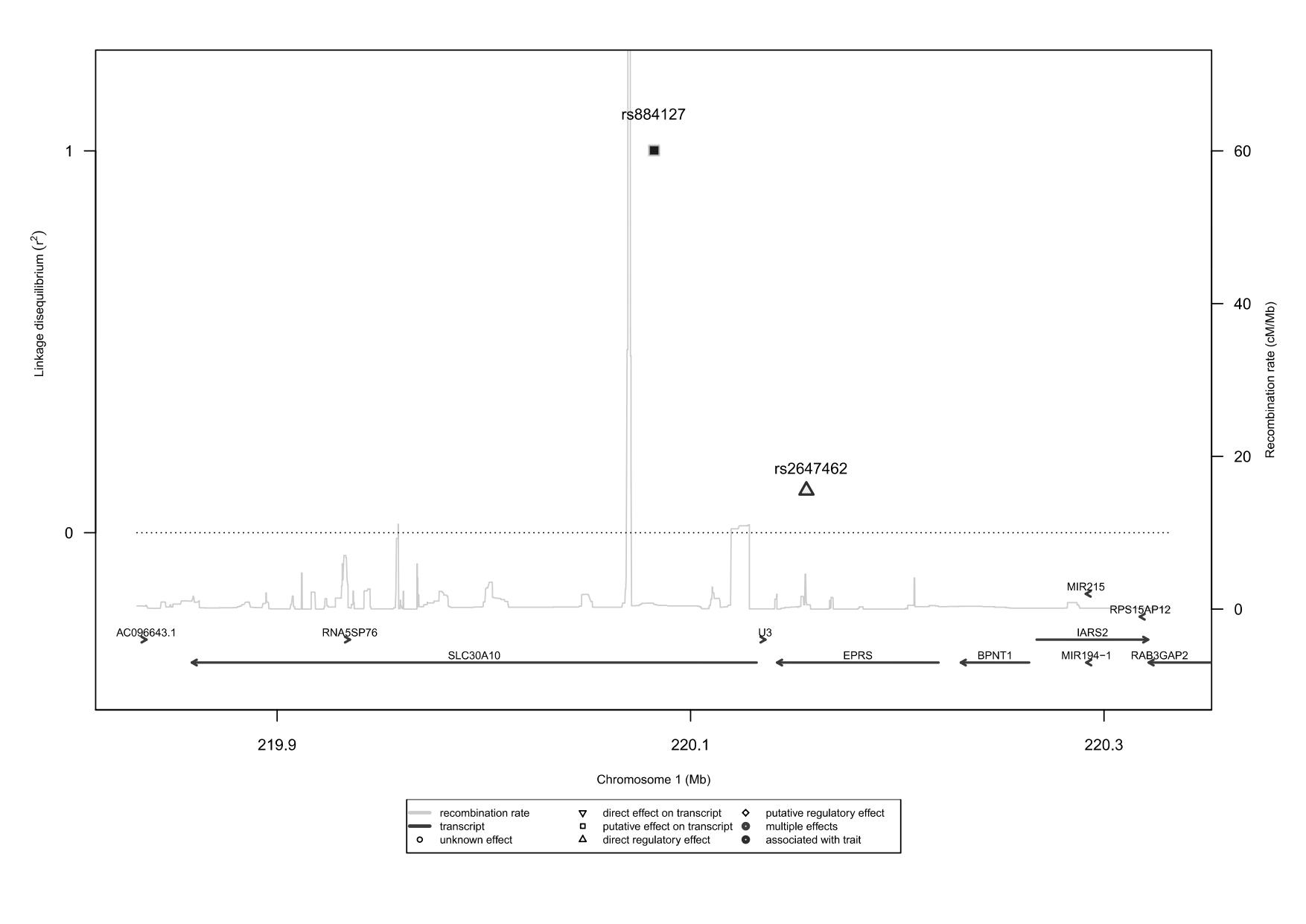

Supplement: Supplementary file 4 — Supplementary Figure 3 [file 41413_2020_101_MOESM4_ESM.jpg]

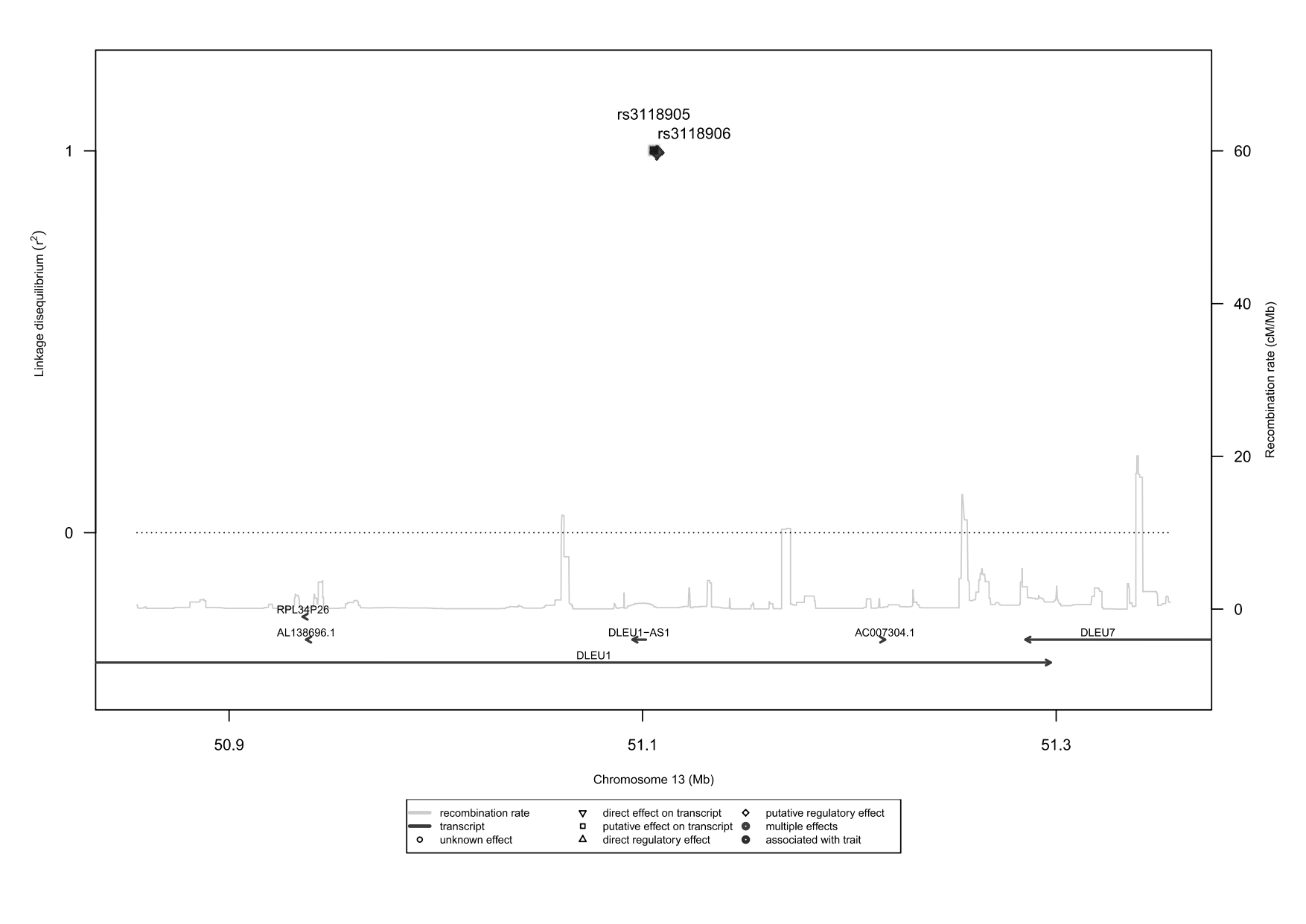

Supplement: Supplementary file 5 — Supplementary Figure 4 [file 41413_2020_101_MOESM5_ESM.jpg]

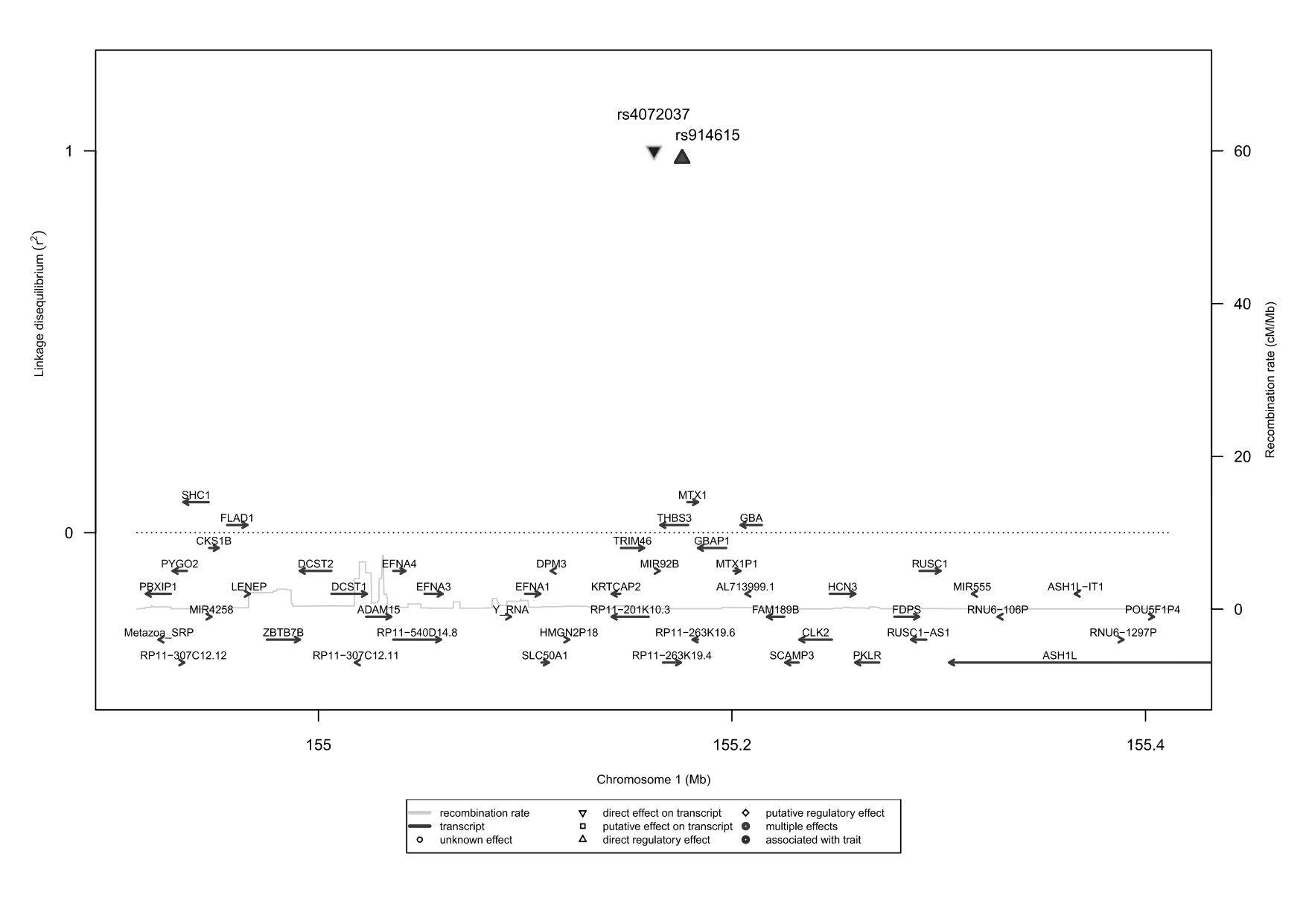

Supplement: Supplementary file 6 — Supplementary Figure 5 [file 41413_2020_101_MOESM6_ESM.jpg]

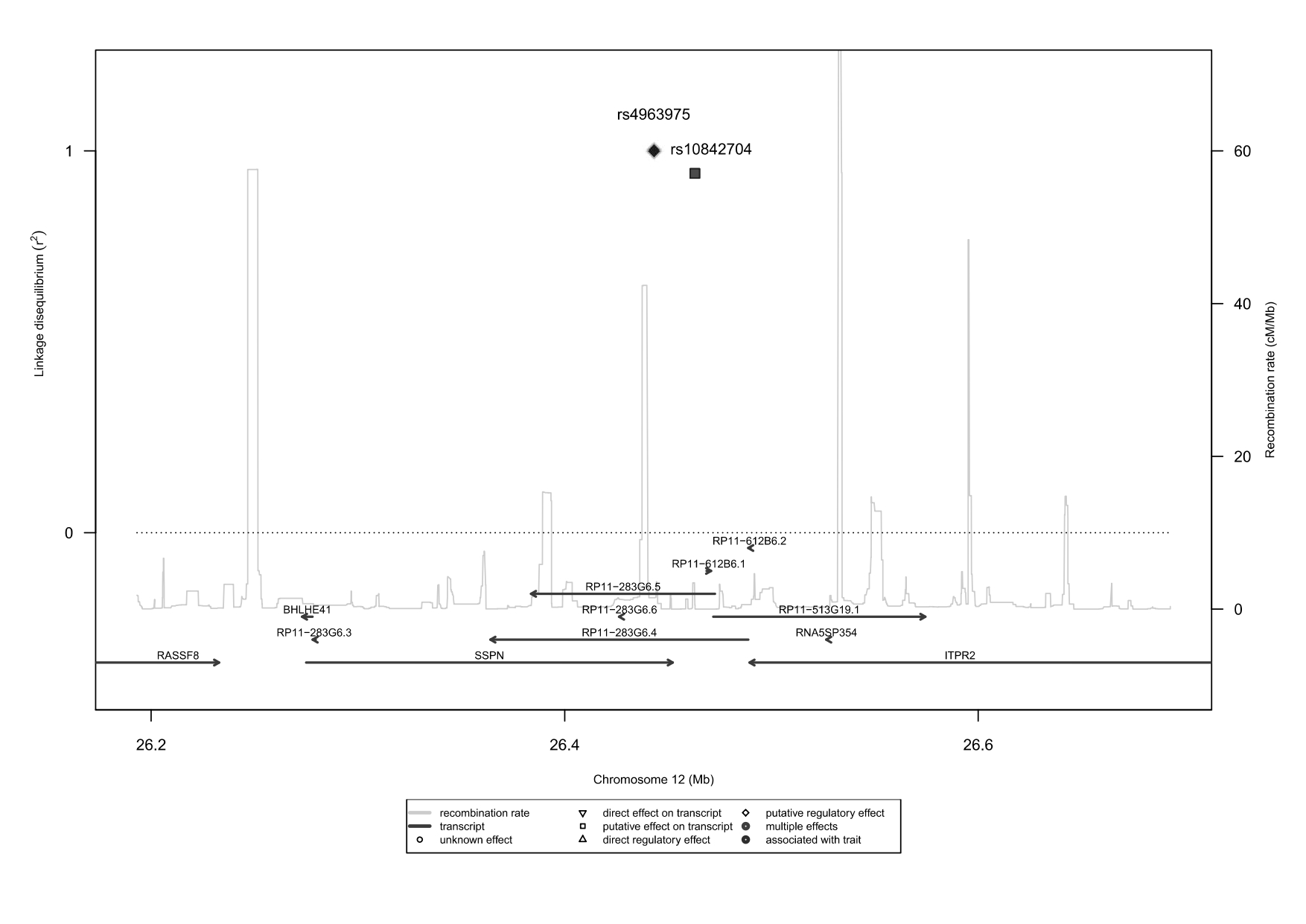

Supplement: Supplementary file 7 — Supplementary Figure 6 [file 41413_2020_101_MOESM7_ESM.jpg]

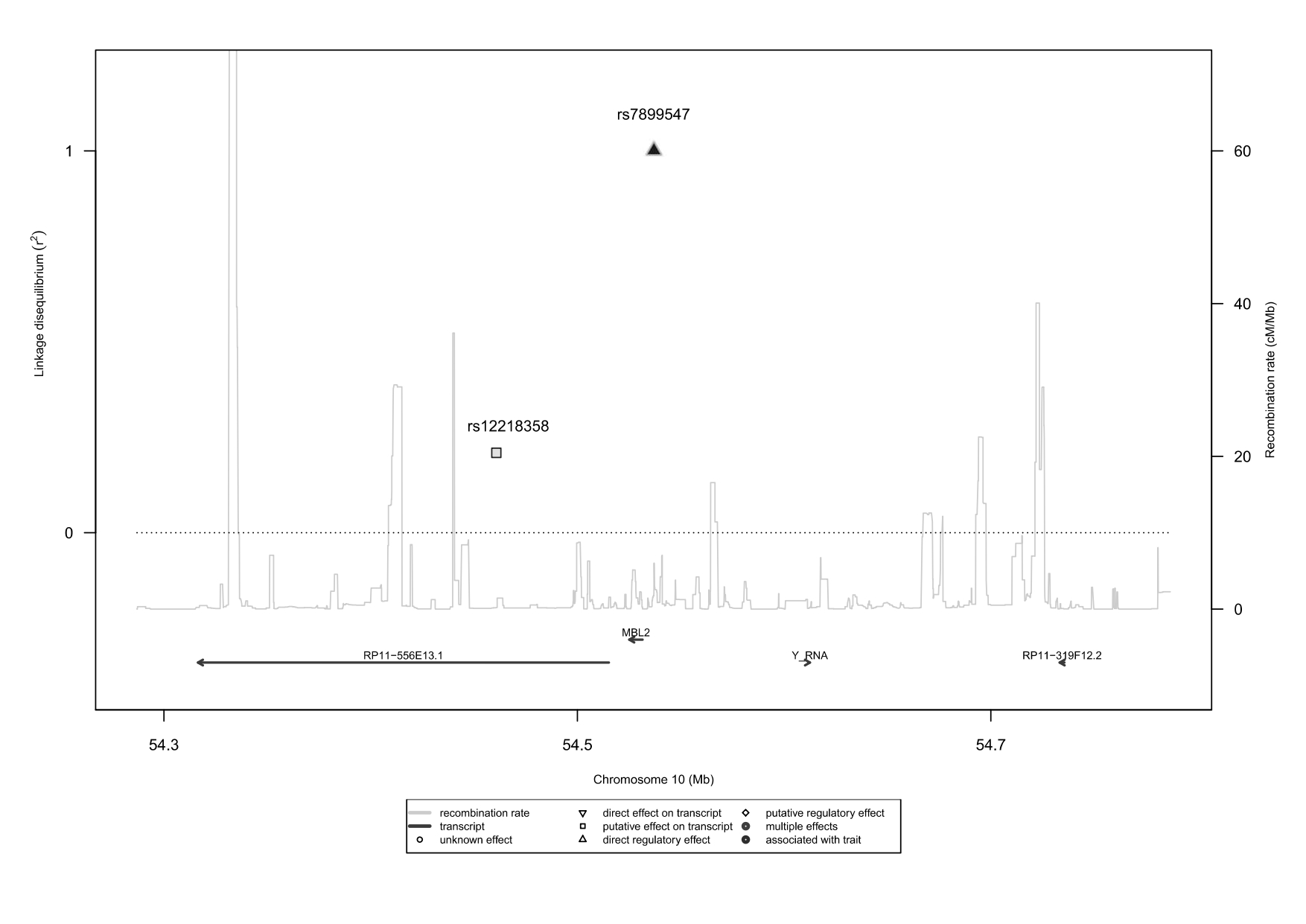

Supplement: Supplementary file 8 — Supplementary Figure 7 [file 41413_2020_101_MOESM8_ESM.jpg]

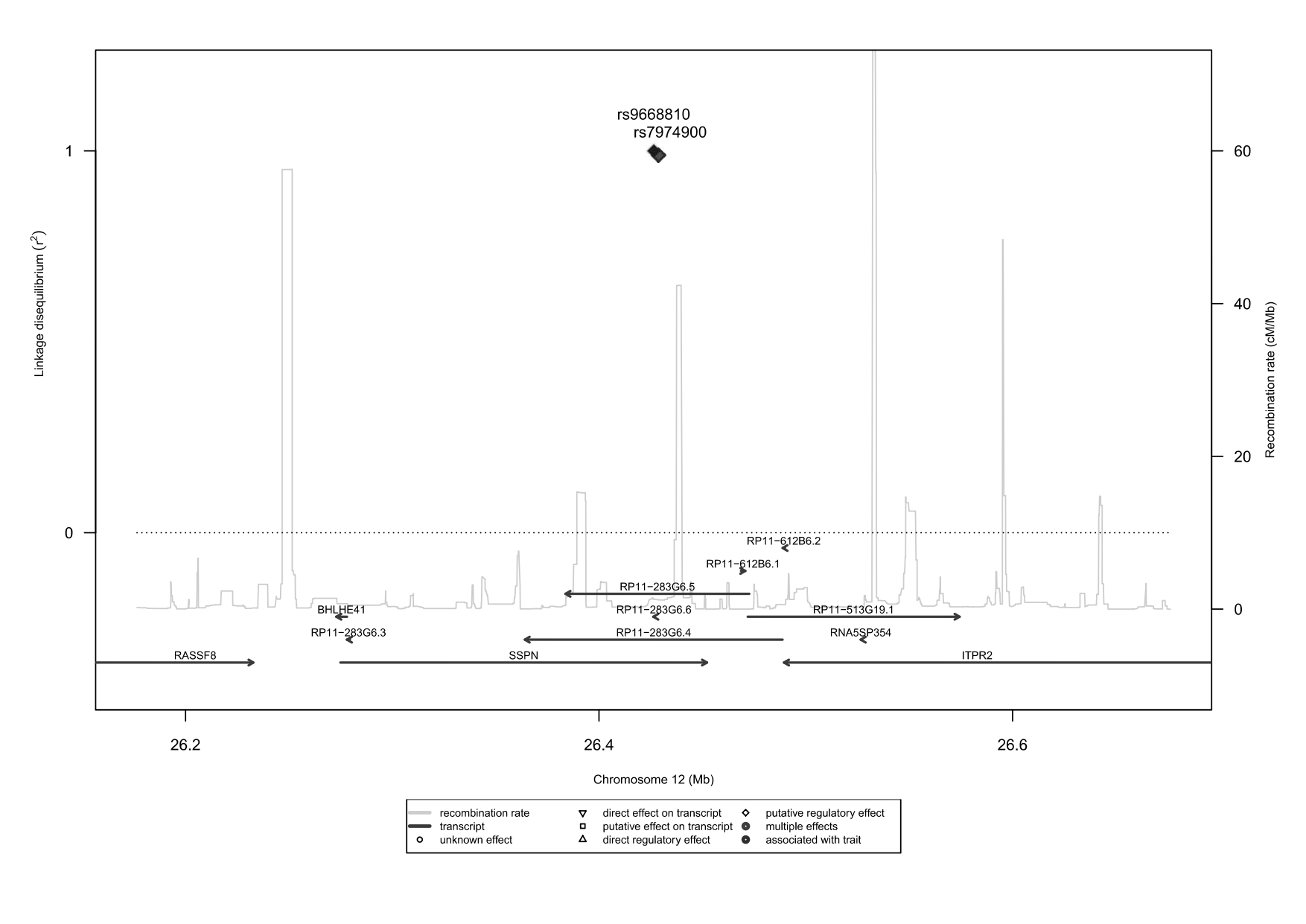

Supplement: Supplementary file 9 — Supplementary Figure 8 [file 41413_2020_101_MOESM9_ESM.jpg]
